# Broad-spectrum inhibition of SARS-CoV-2 variants by dibutyl phthalate through allosteric disruption of Spike-ACE2 interface

**DOI:** 10.3389/fmicb.2025.1610775

**Published:** 2026-01-27

**Authors:** Jiafan Chen, Dekuan Guo, Xiaoxuan Guo, Lijun Zhao, Geng Li, Helu Liu, Shaobo Wang, Zizhao Lao, Meiling Zhu

**Affiliations:** 1 Shenzhen Traditional Chinese Medicine Hospital, Shenzhen, Guangdong, China; 2 Shenzhen Baoan Women’s and Children’s Hospital, Shenzhen, Guangdong, China; 3 State Key Laboratory of Traditional Chinese Medicine Syndrome, Guangzhou University of Chinese Medicine, Guangzhou, China; 4 Shenzhen Clinical College of Integrated Chinese and Western Medicine, Guangzhou University of Chinese Medicine, Shenzhen, Guangdong, China; 5 Chinese Medicine Guangdong Laboratory, Hengqin, Guangdong, China; 6 Guangzhou National Laboratory, Department of Basic Research, Guangzhou International Bio-Island, Guangzhou, China; 7 State Key Laboratory of Respiratory Disease, Guangzhou Laboratory Clinical Base, The First Affiliated Hospital of Guangzhou Medical University, Guangzhou, China

**Keywords:** Dibutyl phthalate, SARS-CoV-2, viral entry inhibitor, Spike-ACE2 interaction, cell membrane fusion, site-directed mutagenesis, structure optimization

## Abstract

**Introduction:**

The persistent evolution of SARS-CoV-2 has diminished the efficacy of existing vaccines and antibodies, increasing the risks of reinfection and Long COVID. There is a significant need for the development of convenient, broad-spectrum antiviral agents that target the early stage of viral infection. Traditional Chinese Medicine (TCM) volatile oils, with their diverse components and suitability for nasal delivery, demonstrate potential against respiratory viruses. This study aimed to screen bioactive compounds from TCM volatile oils for their ability to inhibit the interaction between the SARS-CoV-2 spike (S) protein and its host receptor, ACE2.

**Methods:**

A virtual screening of 47 structurally diverse TCM volatile compounds was performed to identify potential inhibitors of the Spike-ACE2 interaction. The top candidate, dibutyl phthalate (DBP), was further evaluated using in vitro assays including Spike-mediated membrane fusion and pseudovirus infection. Its mechanism was investigated through ELISA, surface plasmon resonance (SPR), ACE2 enzymatic activity assays, molecular docking. To evaluate its broad-spectrum potential, membrane fusion assays were further performed using spike proteins from the wild-type (WT), Delta, and Omicron XBB.1.5 variants. Critical binding residues were identified through molecular docking and subsequently confirmed by site-directed mutagenesis of the Spike receptor-binding domain (RBD).

**Results:**

Virtual screening identified ten potential inhibitors, with dibutyl phthalate (DBP) showing the strongest activity. DBP effectively inhibited S protein-mediated membrane fusion (*IC*_50_ = 64.53 μM) and pseudovirus infection (*IC*_50_ = 73.06 μM) with specificity. SPR analysis confirmed that DBP competitively inhibited the binding between the S trimer and ACE2 (increasing the K_*D*_ from 8.28 nM to 86.7 nM). Mechanistic studies revealed that DBP disrupts the S-ACE2 interaction by targeting the receptor-binding domain (RBD) without affecting ACE2 enzymatic activity. Furthermore, DBP exhibited broad-spectrum inhibitory activity against membrane fusion mediated by the Delta (*IC*_50_ = 49.22 μM) and Omicron XBB.1.5 (*IC*_50_ = 53.70 μM) spike variants. Molecular docking and subsequent site-directed mutagenesis identified Tyr453 and Tyr495 as critical residues for DBP binding and its inhibitory function.

**Discussion:**

This study elucidates for the first time that DBP functions as a broad-spectrum RBD inhibitor. It binds to the RBD-ACE2 interface, dependent on conserved residues Tyr453 and Tyr495, and acts primarily through steric hindrance to block the Spike-ACE2 interaction. Notably, DBP shares critical aromatic and ester groups with other active-site inhibitors. Structure-activity relationship analysis of its derivatives revealed that introducing additional hydrogen-bond acceptors significantly enhances inhibitory activity, providing a clear structure optimization strategy. While DBP has known toxicity, its antiviral potential may be harnessed through strategic delivery approaches or SAR-guided optimization to advance its development against SARS-CoV-2 variants.

## 1 Introduction

SARS-CoV-2 has evolved enhanced immune evasion and transmissibility through frequent genomic mutations (Chakraborty et al., 2022; Zhou et al., 2022). A notable example is the JN.1 variant, which became the dominant strain during recent infection peaks due to adaptive evolution of key spike protein residues, particularly L455S (Jian et al., 2025; Naveed et al., 2025; Tian et al., 2025). Current epidemiological patterns have entered a “new normal” characterized by recurrent small waves of outbreaks (Callaway, 2023), which increase health risks associated with reinfection. A retrospective analysis of data from 74,075 residents in China revealed an exponential increase in the risk of Long COVID-19 (LC) for those who had more than two SARS-CoV-2 infections (OR > 2, FDR < 0.05). The main symptoms include persistent fatigue, cognitive issues, and reduced exercise tolerance (Qin et al., 2024). To address this, antiviral small molecules have become central to prevention and treatment strategies. Approved drugs, such as RNA-dependent RNA polymerase (RdRp) inhibitors (e.g., molnupiravir) and 3CL protease inhibitors (e.g., nirmatrelvir, ritonavir, or leritrelvir), effectively reduce the risk of severe disease by inhibiting viral replication. However, RdRp inhibitors may carry host-mutagenic risks (Zhou et al., 2021), and current treatments primarily target high-risk populations. This leaves a gap in early intervention for mild or asymptomatic cases. Consequently, there is growing interest in developing small molecule drugs that are convenient, home-usable and have broad-spectrum antiviral activity, and target the virus’s early phase. This approach could help prevent infections and treat them earlier.

SARS-CoV-2, which belongs to the *Coronaviridae* family, is an RNA coronavirus family with a high mutation rate. It has four structural proteins: spike, envelope, membrane, and nucleocapsid proteins, as well as 16 non-structural proteins. The spike (S) protein mediates viral invasion by binding to the host ACE2 receptor through its receptor-binding domain (RBD). This process occurs in three stages: (1) Receptor binding: the RBD of the Spike protein binds to ACE2 with high affinity, allowing the virus to attach to host cells (Wang et al., 2021); (2) Plasma membrane fusion: Host proteases Furin (Hoffmann et al., 2020a) and TMPRSS2 (Hoffmann et al., 2020b) cleave the Spike protein at the S1/S2 and S2’ sites, respectively, triggering conformational changes in the S2 subunit that expose the fusion peptide (FP) to directly mediate fusion between the viral and cellular membranes; Alternatively, (3) Endocytosis: if membrane fusion fails, the virus enters the cell via clathrin-mediated endocytosis. In this pathway, endosomal proteases—primarily cathepsins L and B—process the Spike protein to activate viral entry into the cytoplasm (Prasad et al., 2021). Among them, the Spike-ACE2 interaction is a crucial initial step and an essential component of the SARS-CoV-2 infection process. Despite different SARS-CoV-2 variants may bind to ACE2 with varying strength, this interaction is still crucial for viral invasion (Liu et al., 2022). In addition to SARS-CoV-2, SARS-CoV, HCoV-NL63, and several closely related bat coronaviruses rely on the Spike-ACE2 interaction to attach to host cells (Letko et al., 2020; Li et al., 2003). Notably, Spike-induced ACE2 shedding or downregulation may contribute to the development of acute respiratory distress syndrome (ARDS) by disrupting the renin-angiotensin-aldosterone system (RAAS) (Vasanthakumar, 2020). Because of its central role in infection, targeting the Spike-ACE2 interaction is considered a promising strategy for preventing SARS-CoV-2 infections, blocking transmission, and reducing associated pathological damage. While existing campaigns have yielded instructive small-molecule and peptide inhibitors (Xiang et al., 2021), the systematic exploration of traditional medicinal volatile oils as a source of drug-like inhibitors remains comparatively limited. This inherent structural diversity and suitability for inhalation delivery of volatile components, offers a compelling alternative avenue for early intervention therapy.

Aromatherapy, originating in ancient Egypt, China, and India circa 3,000 BC, represents a traditional medical practice utilizing plant-derived volatile oils for disease prevention and treatment (Koyama and Heinbockel, 2020). The *Shennong Bencao Jing* (*Classic of Herbal Medicine*) documents the epidemic-prevention properties of aromatic herbs including Artemisia argyi and Atractylodes rhizomes, while the *Bencao Gangmu* (*Compendium of Materia Medica*) systematically elucidates the mechanisms by which aromatic plants achieve “pathogen elimination and impurity removal” through fumigation. Modern research has revealed that volatile oils constitute complex mixtures of diverse small-molecular compounds, including monoterpenes, sesquiterpenes, alcohols, aldehydes, ketones, and esters (de Groot and Schmidt, 2016). These volatile and biocompatible components demonstrate potential in the prevention and treatment of respiratory viral infections through nasal administration, enabling rapid absorption via respiratory mucosa (Oriola and Oyedeji, 2022). Notably, patchouli alcohol from Patchouli oil exhibits anti-influenza activity by inhibiting viral neuraminidase (Wu et al., 2011), while eucalyptus oil and its primary component 1, 8-cineole significantly reduce SARS-CoV-2 spike pseudovirus infectivity (Fernandez et al., 2024). Based on this evidence, we hypothesize that specific components in traditional Chinese medicinal volatile oils can inhibit SARS-CoV-2 Spike-ACE2 interaction.

To validate this hypothesis, this study screened natural products from essential oils of TCM with anti-upper respiratory tract virus activity. The findings revealed that DBP could reduce viral adsorption efficiency by blocking the RBD-ACE2 interaction, inhibit spike-mediated membrane fusion, and suppress pseudovirus infection. Critically, site-directed mutagenesis demonstrated that the antiviral activity of DBP is dependent on key residues Tyr453 and Tyr495 of the RBD. Recent reviews indicate that although phthalate esters are traditionally recognized as plasticizers with some toxicity, structurally similar bioactive natural products are widely produced in living systems, including algae, bacteria, and higher plants (Roy, 2020). Research has shown that novel phthalate acid esters derived from *Acrostichum aureum* L., a traditional medicinal plant used for treating infections, along with cellulose acetate phthalate (CAP), exhibit efficacy against several viruses, including dengue virus, human parainfluenza virus, and chikungunya virus (Uddin et al., 2013). CAP has also demonstrated anti-HIV activity by targeting co-receptor sites and anti-HSV properties in multiple studies (Manson et al., 2000; Subhra et al., 2017; Huang et al., 2012; Yun-Yao et al., 2001). Additionally, cyclic dimer phthalate derivatives have shown potential in combating Hepatitis C virus and SARS-CoV-2 (Saito et al., 2022). Di-n-octyl phthalate, found in the methanol extract of Syzgium cumini, could serve as a future antiviral agent, particularly for direct-acting antiviral therapy against the Hepatitis C virus. Thus, phthalate derivatives, including DBP, are promising antiviral compounds, with DBP formed by linking two butyl groups to the phthalate moiety. Although DBP exhibits certain toxicity, its lipophilic nature may allow for targeted delivery via localized administration, such as nasal spray, thereby reducing the required dosage and minimizing side effects. Additionally, combining DBP with conventional small-molecule drugs or optimizing its structure through linker design could further enhance its antiviral activity.

## 2 Results

### 2.1 Virtual screening of aromatic compounds that inhibit ACE2 and SARS-CoV-2 Spike interaction

**FIGURE 1:**
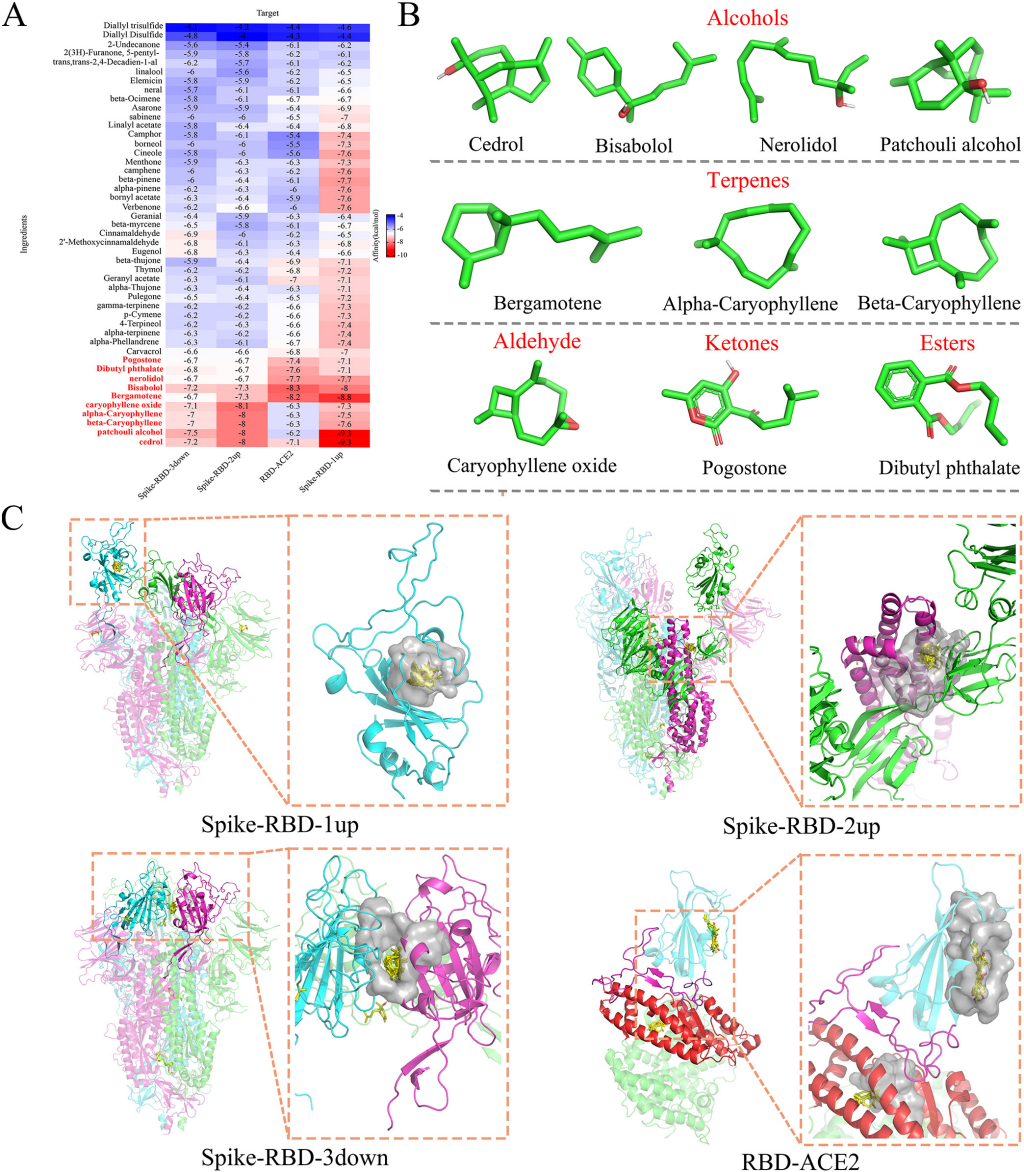
Virtual screening of aromatic compounds effectively inhibiting ACE2-Spike interaction. **(A)** The heatmap of molecular docking structures of 47 aromatic compounds with different con-formations of Spike and ACE2. The color represents the docking binding energy, with a redder color indicating a more stable binding capacity. The top 10 compounds (indicated in red) were utilized for subsequent experimental detections. **(B)** Structure and classification of 10 potentially effective compounds. **(C)** The protein pockets (in gray) where the top 10 compounds (in yellow) bind to different conformations of Spike and ACE2. Spike-RBD-1up/Spike-RBD-3down: RBD is highlighted in solid color; Spike-RBD-2up: The S1 and S2 subunits involved in the pocket are emphasized in solid color; RBD-ACE2: RBD is presented in blue, ACE2 in green, the RBM se-quence in direct contact with ACE2 is in pink, and the interface in direct contact with RBD on ACE2 is in red.

Forty-seven natural products, sourced from TCM essential oils with anti-upper respiratory tract virus activity, were collected (Supplementary Table 1). To identify compounds that potentially inhibit the binding of ACE2 to the SARS-CoV-2 spike (Spike) protein, virtual screening was conducted using various cryo-electron microscopy structures of the S-protein and ACE2 during the SARS-CoV-2 entry process. These structures included 7TL9 (with the receptor-binding domain (RBD) in chain A in the “up” open conformation), 8C5R (with RBDs in chains A and B in the “up” open conformation), 7TF8 (with all RBDs in the “down” closed conformation), and 7XWA (a complex structure where RBDs are bound to ACE2). Based on the molecular docking binding energies (Supplementary Table 2), hierarchical clustering analysis was performed on a heatmap (Figure 1A) to identify the top 10 compounds with the best binding performance. These compounds included four alcohols, three terpenoids, and one compound each of ethers, ketones, and esters (Figure 1B). These compounds were further analyzed for potential binding pockets in the target proteins.

In the Spike-RBD-1up conformation (Figure 1C), the compounds primarily stabilize a protein pocket located below the receptor-binding motif (RBM). These compounds may allosterically affect the RBD’s conformational changes, potentially decreasing its stability and reducing binding to ACE2. In the Spike-RBD-2up conformation, the compounds mainly stabilize the protein pocket formed by the CT1 (528–590) region of S1, S2’ (686–815) of S2, and Heptad Repeat 1 (HR1) (912–984), located near the fusion peptide (FP) region (816–855). These compounds may influence the release of the HR1 hinge, thereby inhibiting viral fusion (Xing et al., 2025). In the Spike-RBD-3down conformation, the compounds occupy a protein pocket between two RBDs in the closed conformation. This pocket contains a fatty acid (FA) binding site that can be stabilized by linoleic acid (LA), potentially locking the Spike protein in the closed conformation, reducing its interaction with ACE2, and inhibiting viral binding (Toelzer et al., 2020). In the ACE2-RBD complex, two binding sites are identified, one at the interface between RBD and ACE2. This may affect the stability of the ACE2-RBD interaction. These data suggest that the 10 identified compounds have the potential to block SARS-CoV-2 infection.

### 2.2 DBP demonstrates the most potent inhibition of ACE2/RBD binding and Spike-mediated membrane fusion

**FIGURE 2:**
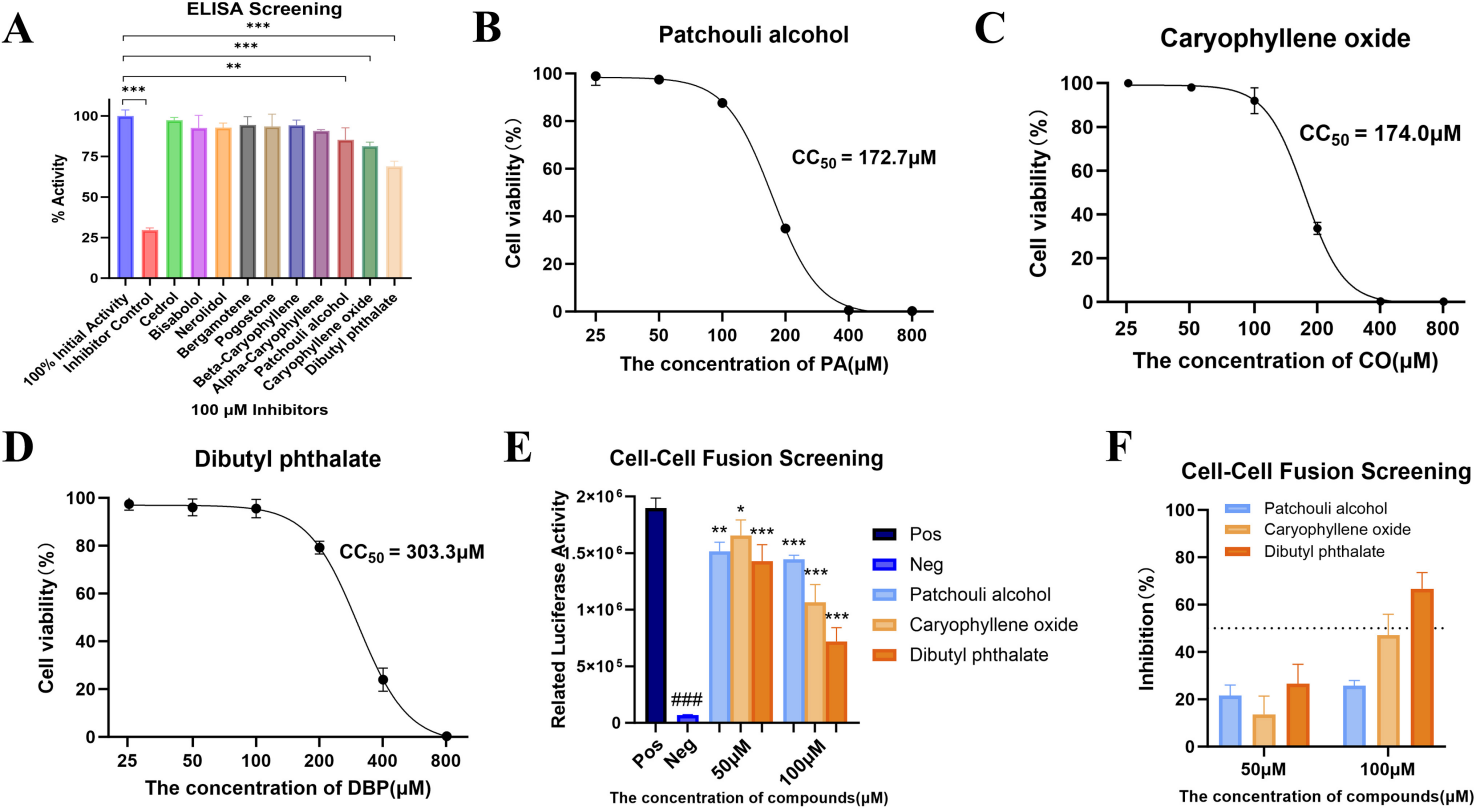
Further screening of potential inhibitors and evaluation of their effects. **(A)** ELISA was performed to evaluate the inhibitory binding activity of 10 potential compounds. Data are means and Standard Deviation (SD) (*n* = 3). Statistical significance between groups was determined using One-way analysis of variance (ANOVA). Comparisons to the 100% initial activity group are de-noted as follows: *P < 0.05; **P < 0.01; ***P < 0.001. **(B–D)** CCK8 assay was used to assess the cytotoxicity of PA, CO, and DBP in Human Embryonic Kidney 293T cells (HEK293T) cells. Data are presented as the mean ± SD from three independent experiments. **(E,F)** Cell membrane fusion assay was performed to evaluate the inhibitory effect of the compounds at concentrations of 50 and 100 μM. Statistical significance of relative luciferase activity values was assessed as follows: ###*P* < 0.001 compared to the positive control group; *P < 0.05, **P < 0.01, ***P < 0.001 compared to the negative control group.

To further screen for effective compounds, the inhibitory activities of ten potential compounds on Spike-ACE2 binding were initially evaluated through Enzyme-Linked Immunosorbent Assay (ELISA) experiments. The outcomes revealed that Patchouli alcohol (PA), Caryophyllene oxide (CO), or Dibutyl phthalate (DBP) significantly inhibited the binding of Spike with ACE2 (Figure 2A). Subsequently, the cytotoxicity of these three compounds was evaluated using the Cell Counting Kit-8 (CCK8) assay (Figures 2B–D). The results demonstrated that the 50% cytotoxic concentration (*CC*_50_) of PA was 172.7 μM, CO exhibited a *CC*_50_ of 174.0 μM, and DBP showed a *CC*_50_ of 303.3 μM. Next, to quantify Spike-mediated membrane fusion, a Cre-LoxP firefly luciferase (Stop-Luciferase) system was employed to detect DNA recombination events during cell-cell fusion, as previously reported. Through the cell membrane fusion assays (Figures 2E, F), the inhibitory effects of the compounds at concentrations of 50 and 100 μM were tested. Eventually, DBP was identified as the most effective inhibitor of cell membrane fusion, with an inhibitory rate of 66.727 ± 5.590% at a concentration of 100 μM.

### 2.3 DBP inhibits SARS-CoV-2 Spike-Mediated cell membrane fusion and SARS-CoV-2 pseudovirus infection

**FIGURE 3:**
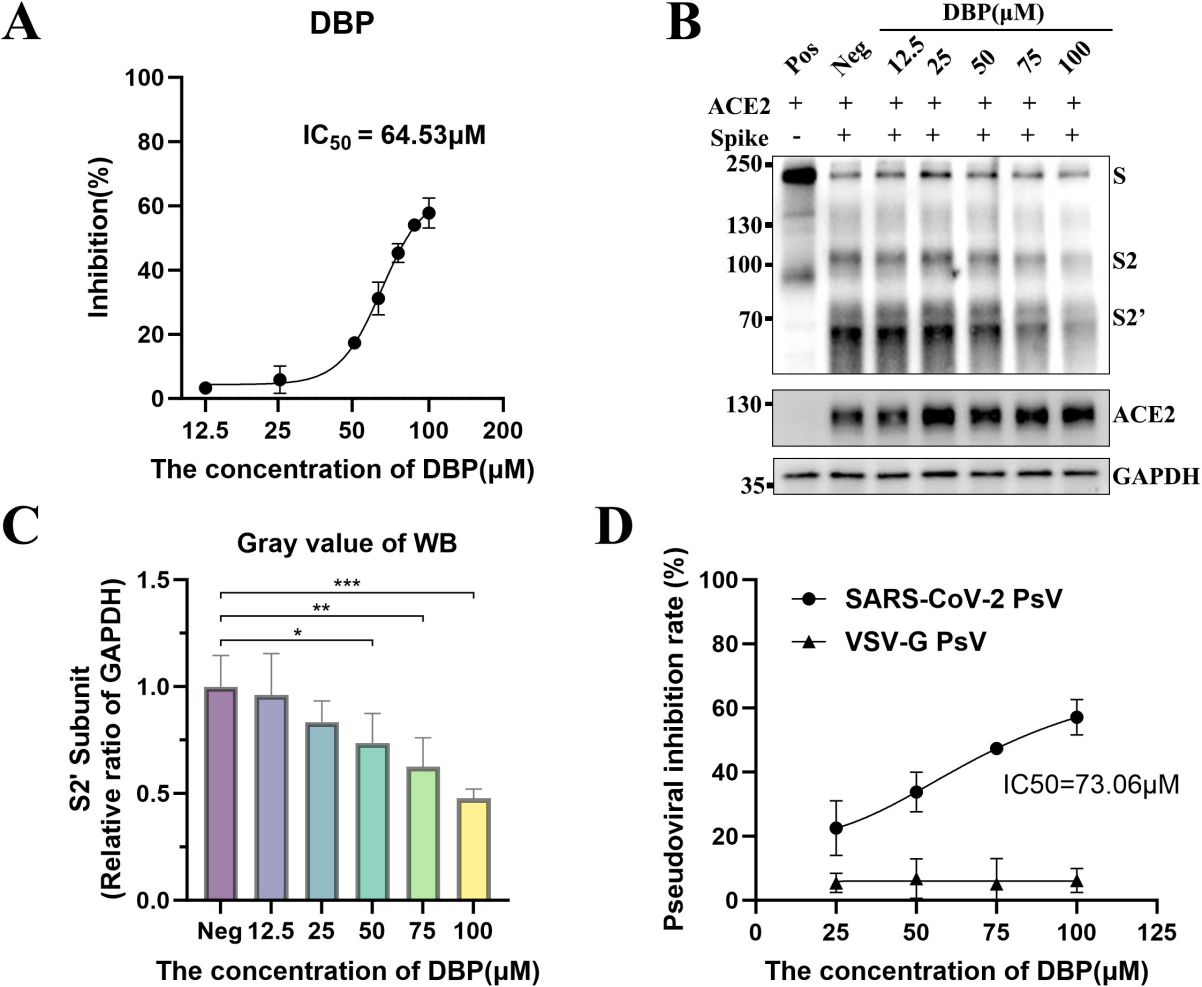
Inhibition of SARS-CoV-2 Spike-mediated cell fusion and PsV infection by DBP. **(A)** DBP inhibits SARS-CoV-2 Spike-mediated cell membrane fusion. Seven different concentrations were tested. **(B)** Western blot analysis of Spike protein cleavage. As DBP concentration increases, the concentration of the S2’ subunit decreases compared to the Neg group. **(C)** Gray scale values of the S2’ subunit from three repeated experiments. The gray value of the S2’ protein strip was reported as mean ± SD. Statistical significance relative to the Neg group is denoted as follows: *P < 0.05, **P < 0.01, ***P < 0.001. **(D)** DBP inhibits SARS-CoV-2 PsV infection but not VSVΔG/G PsV.

To assess DBP’s inhibitory effect on SARS-CoV-2 Spike-mediated cell membrane fusion and SARS-CoV-2 PsV infection, we initially performed a cell membrane fusion experiment. In this experiment, effector cells expressing the SARS-CoV-2 S-WT protein and Cre recombinase (293T/SARS-CoV-2-S-WT/Cre) were co-cultured with target cells expressing the hACE2 receptor and the Stop-luc reporter gene (293T/hACE2/Stop-luc). The results indicated that DBP had a 50% inhibitory concentration (*IC*_50_) of 64.53 μM for cell membrane fusion (Figure 3A). Furthermore, we assessed Spike protein cleavage using Western blot (Figure 3B). The results demonstrated that the DBP downregulated the expression of the S2’ subunit in a dose-dependent manner, suggesting that DBP significantly inhibited the fusion between effector cells and target cells. Figure 3C presents the gray values of the S2’ subunit based on three replicated experiments. In the 100 μM DBP treatment group, the gray value of the S2’ subunit was 0.520 ± 0.029, which was lower than the Neg group. Subsequently, we evaluated the inhibitory effect on viral infection with a SARS-CoV-2 pseudovirus experiment. The *IC*_50_ of DBP on SARS-CoV-2 pseudovirus was 73.06 μM (Figure 3D). we also tested DBP’s effect VSVΔG/G pseudovirus infection. The results indicated that DBP did not significantly inhibit VSVΔG/G pseudovirus infection, further confirming the specificity of DBP for SARS-CoV-2 pseudovirus.

### 2.4 Fluorescence co-localization reveals DBP inhibition of Spike-mediated membrane fusion

**FIGURE 4:**
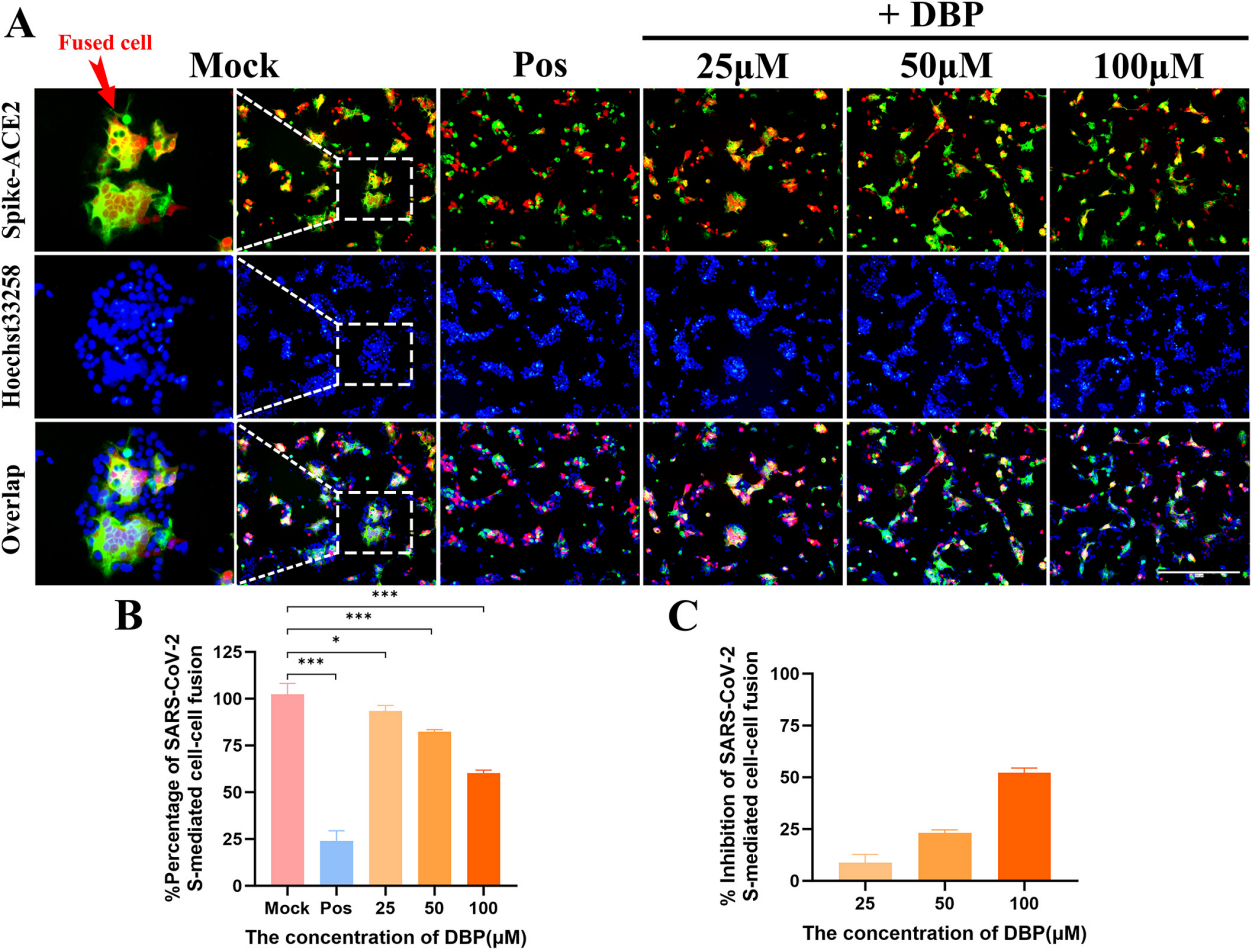
Inhibition of SARS-CoV-2 Spike-mediated cell membrane fusion by DBP as assessed by fluorescence co-localization. **(A)** Fluorescent images were captured at 24 h after treatment with DBP to assess SARS-CoV-2 Spike-mediated cell-cell fusion. The green and red fluorescence signals are merged, and the yellow cells represent fused cells (red arrow). The blue fluorescence signal represents the nucleus. **(B)** Percentage of SARS-CoV-2 Spike-mediated cell-cell fusion. The ratio of yellow (fusion cells) to blue (total cells) areas was calculated from the fluorescent images. The membrane fusion percentage was reported as mean ± SD. Statistical significance is denoted as follows: *P < 0.05, **P < 0.01, ***P < 0.001 versus the Mock group. **(C)** Statistical analysis of the membrane fusion inhibition rate. Three randomly selected fields per sample were analyzed from each sample.

To observe the inhibitory effect of DBP on cell membrane fusion, a fluorescence colocalization assay was employed to evaluate the quantity of membrane-fused cells after DBP treatment. In this experiment, 293T cells in the Mock group and the experimental group were co-transfected with mCherry and ACE2 plasmids (293T/hACE2/mCherry). mCherry serving as a marker for ACE2, appearing red. Simultaneously, another group of 293T cells was singly transfected with Spike plasmids containing a GFP tag (293T/SARS-CoV-2-Spike/GFP), appearing green. When ACE2 and Spike bind, membrane fusion occurs, forming yellow fused cells (Figure 4A). In contrast, the positive control group lacked hACE2 plasmid, so no yellow fused cells were observed. Hoechst 33,528 dye was utilized to label the cell nuclei as blue for cell enumeration. The results revealed that in comparison with the Mock group, the positive control group had almost no yellow fused cells. In the experimental group, as DBP concentration increased, the number of membrane-fused cells decreased significantly. Subsequently, the ratio of the yellow to the blue regions (yellow/blue) in the fluorescence images was calculated. Results from three replicate experiments were analyzed statistically. The percentage of fused cells and the inhibition rate of membrane fusion are depicted in Figures 4B, C. At 100 μM, DBP inhibited 53.201 ± 1.712% of Spike-mediated membrane fusion.

### 2.5 Surface plasmon resonance elucidates the impact of DBP on Spike-ACE2 interactions

**FIGURE 5:**
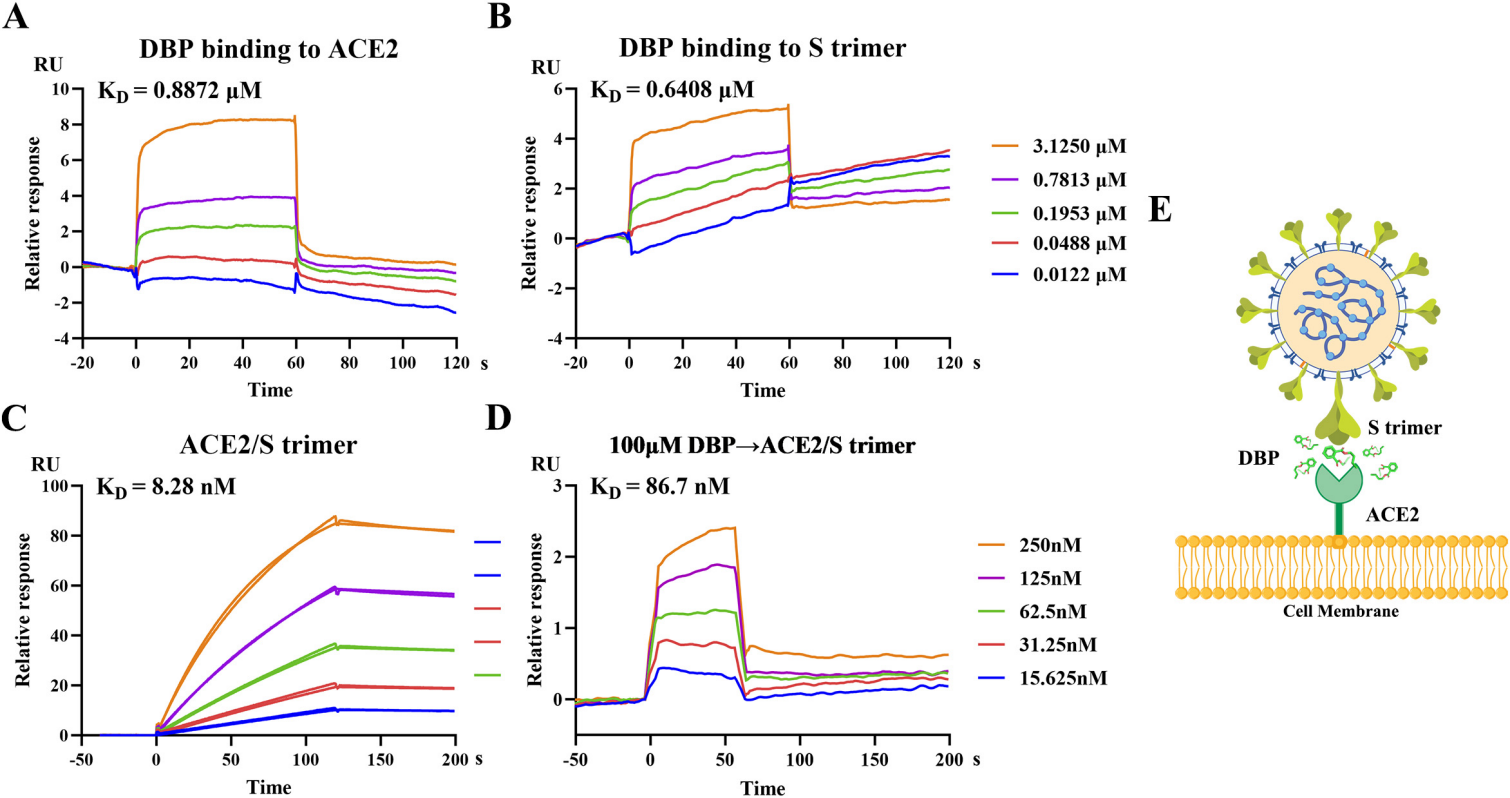
SPR Analysis of DBP Binding to ACE2 and SARS-CoV-2 Spike Trimer, and Its Inhibition of Spike-ACE2 Interaction. **(A,B)** The response curves of DBP (0.0122–3.1250 μM) with ACE2 (40 μg/mL, optimized for DBP-ACE2 binding detection) and S trimer (40 μg/mL, optimized for DBP-S trimer binding detection). **(C)** Concentration-dependent binding of ACE2 (15.625–250 nM) to S trimer (20 μg/mL, optimized for ACE2-S trimer binding detection). **(D)** Inhibitory effect of DBP on S trimer-ACE2 interaction. K_D_: Equilibrium dissociation constant. **(E)** Proposed mechanism of action of DBP.

SPR experiments were conducted to examine DBP’s affinity for both the SARS-CoV-2 spike protein trimer (S trimer) and ACE2, as well as its effect on the Spike-ACE2 interaction. DBP demonstrated high affinity for both proteins, with equilibrium dissociation constant (K_*D*_) values of 0.640 μM for the S trimer and 0.887 μM for ACE2 (Figures 5A,B). Without DBP, the K_*D*_ of ACE2 for the S trimer was 8.28 nM. However, when 100 μM DBP was added, this value increased significantly to 86.7 nM. Additionally, the maximum response (Rmax) decreased from 108 to 3.6 at 250 nM ACE2 in the presence of DBP (Figures 5C, D). These findings suggest that DBP competitively inhibits the S trimer-ACE2 interaction, which is important for viral attachment and host cell entry. A summary of the SPR kinetic parameters is shown in Table 1. The proposed mechanism of DBP action is summarized in Figure 5E.

**TABLE 1:** SPR kinetic parameters.

Interaction	KD	Kon (1/Ms)	Koff (1/s)	U-value	Rmax (RU)	offset (RU)	Chi^2^ (RU^2^)
DBP→ACE2	0.8872 μM	/	/	/	11.16	–0.6368	0.931
DBP→S trimer	0.6408 μM	/	/	/	4.139	1.601	0.306
ACE2/S trimer	8.28 nM	5.45E + 04	4.51E-04	7	108	/	0.649
DBP→ACE2/S trimer	86.7 nM	/	/	/	3.6	–0.2	0.000602

### 2.6 DBP inhibits SARS-CoV-2 RBD-ACE2 interaction without affecting ACE2 enzymatic activity

**FIGURE 6:**
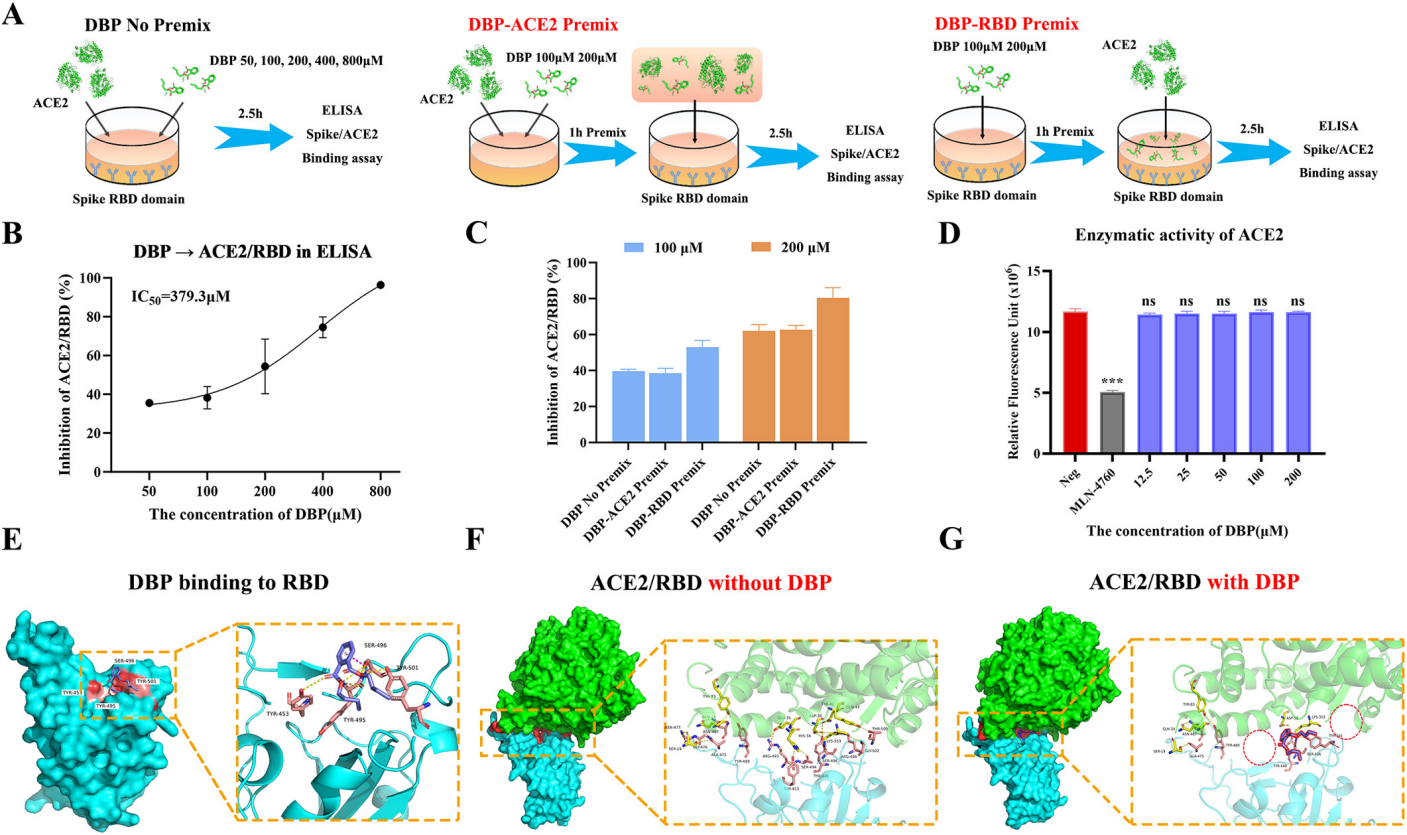
Inhibition of SARS-CoV-2 RBD-ACE2 interaction by DBP and its effect on ACE2 enzymatic activity. **(A)** Schematic illustration of ELISA assays under three experimental conditions: DBP No Premix, DBP-ACE2 Premix, and DBP-Spike Premix. **(B)** ELISA showing the inhibitory effect of DBP on the binding of SARS-CoV-2 RBD to ACE2. **(C)** Bar graph depicting the inhibition rate of ACE2/RBD binding by DBP under varied preincubation conditions. DBP at concentrations of 100 μM (blue) and 200 μM (orange) was evaluated in three conditions: no preincubation (DBP No Premix), preincubation with ACE2 (DBP-ACE2 Premix, 1 h), and preincubation with RBD (DBP-RBD Premix, 1 h). **(D)** Assessment of DBP’s effect on ACE2 enzymatic activity within a concentration range of 12.5–200 μM. Relative Fluorescence units were reported as mean ± SD. Significant differences were observed in the MLN-4760 group compared to the Neg group (***P < 0.001), while no statistically significant differences (ns) were detected in the other experimental groups. **(E)** Molecular docking analysis showing that DBP stably binds at the RBD (Cyan). **(F)** Structural representation of the ACE2-RBD interface (Red) before DBP binding, showing the formation of 18 hydrogen bonds (Yellow) and one salt bridge (Orange). ACE2 inter-action residues are shown in yellow, RBD residues in salmon. **(G)** Structural representation of the ACE2-RBD interface (Red) after DBP binding, with only 7 hydrogen bonds remaining.

The SARS-CoV-2 S trimer binds to the host ACE2 receptor through its RBD. To assess the inhibitory effect of DBP on this binding, we used ELISA, which showed an *IC*_50_ of 379.3 μM for DBP (Figures 6A, B). To determine whether DBP acts on ACE2 or RBD, we designed three preincubation conditions in the ELISA assay (Fu et al., 2021): no preincubation (DBP No Premix), preincubation of DBP with ACE2 (DBP-ACE2 Premix, 1 h), and preincubation of DBP with RBD (DBP-RBD Premix, 1 h) (Figure 6A). Specifically, preincubating DBP with RBD resulted in a more pronounced inhibitory effect on ACE2-RBD binding compared to preincubation with ACE2 or no preincubation (Figure 6C), indicating that DBP primarily targets RBD to disrupt the interaction.

Given ACE2’s roles in regulating blood pressure, reducing inflammation, and protecting heart function (Li et al., 2024), we further examined DBP’s impact on ACE2 enzyme activity. DBP had no significant effect on ACE2 activity at concentrations between 12.5 and 200 μM, while the positive control, MLN4760, significantly decreased ACE2 activity (Figure 6D). Molecular docking revealed that DBP stably binds at the RBD-ACE2 interface (Figure 6E), where the “head” (benzene ring) forms a π-cation bond with SER-496, and the oxygen atoms of the “legs” (ester groups) form hydrogen bonds with TYR-501, Tyr495, and SER-496. Additionally, one “hand” (acyl group) forms a hydrogen bond with Tyr453. We further analyzed the ACE2/RBD interaction, both with and without DBP, using the HDOCK server (Figures 6F, G). The results showed a decrease in hydrogen bonds from 18 to 7 and the loss of the salt bridge (GLU-35, ARG-493). The binding score increased from –368.24 to –270.71 (Table 2). Protein residues marked with an asterisk (*) in Table 2 indicate those that lost their interaction after DBP binding. In general, a lower (more negative) docking score reflects a stronger binding affinity. Overall, DBP effectively disrupts the RBD-ACE2 interaction without affecting ACE2’s enzymatic activity.

**TABLE 2:** Protein-protein docking analysis.

Docking system	Docking score	The residues of ACE2	The residues of Spike RBD
ACE2/RBD without DBP	–368.24	SER-19, GLN-24, HIS-34*, GLU-35*, ASP-38, TYR-41*, GLN-42*, TYR-83, LYS-353	TYR-449, Tyr453*, ALA-475, GLY-476*, ASN-477*, ASN-487, TYR-489, ARG-493*, SER-494*, SER-496, ARG-498*, THR-500*, GLY-502*
ACE2/RBD with DBP	–270.71	SER-19, GLN-24, ASP-38, TYR-83, LYS-353	TYR-449, ALA-475, ASN-487, TYR-489, SER-496, TYR-501

*The residues lost their interaction after DBP binding.

### 2.7 Inhibition of DBP on SARS-CoV-2 mutants

**FIGURE 7:**
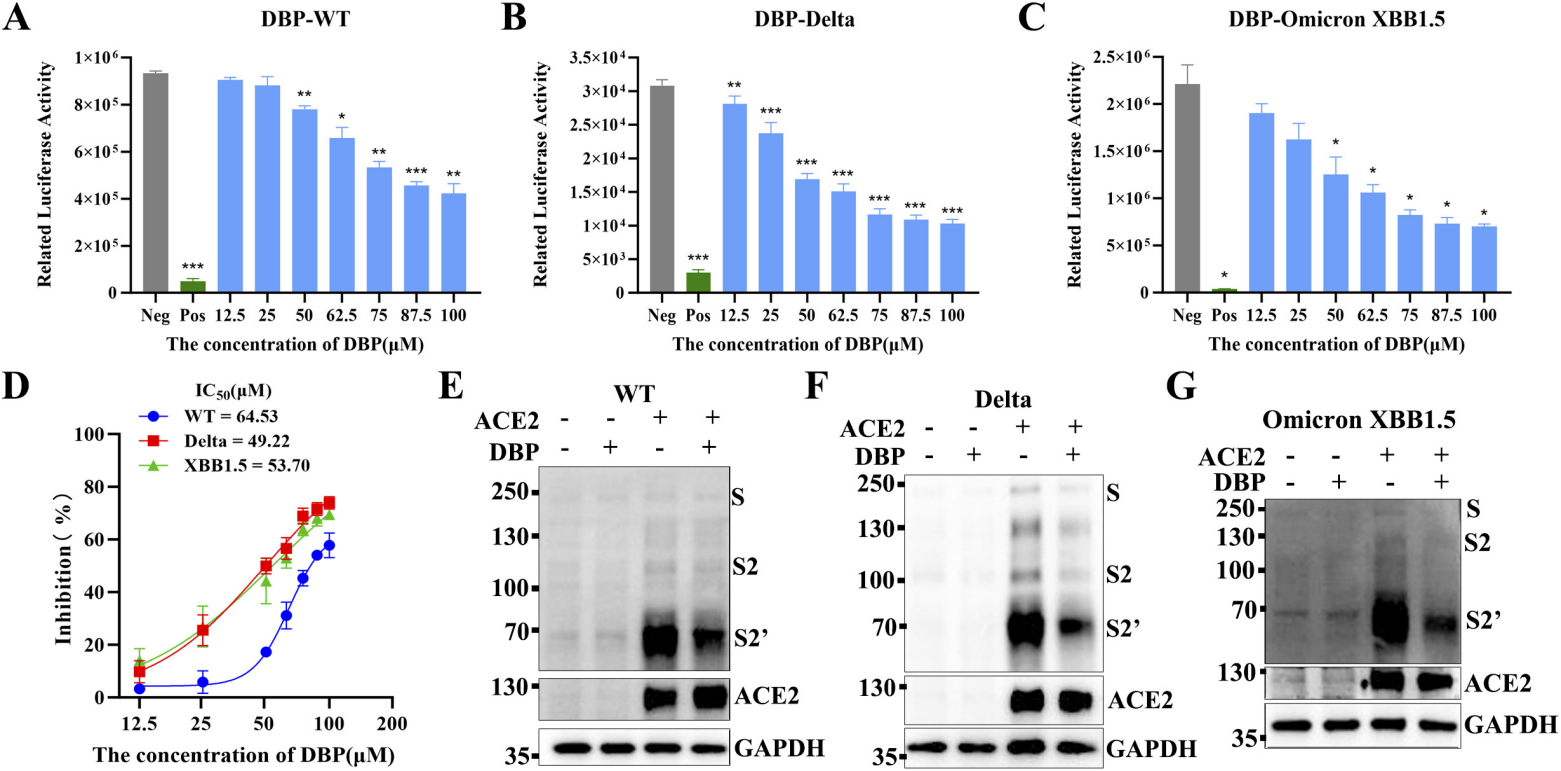
Inhibition of Spike-mediated membrane fusion by DBP in SARS-CoV-2 WT, Delta and Omicron XBB1.5 variants. **(A–C)** The luciferase expression after cell membrane fusion. Membrane fusion activity in cell lysates was quantified using a luciferase reporter assay. Data are presented as mean ± SD. Statistical significance is denoted as follows: *P < 0.05, **P < 0.01, ***P < 0.001, versus the Neg group. **(D)** Dose-response curve showing the *IC*_50_ of DBP. **(E–G)** Western blot analysis showing the inhibition of Spike-mediated membrane fusion.

To investigate DBP’s inhibitory effect on various SARS-CoV-2 mutant strains, membrane fusion experiments were performed using spike proteins from the Delta and Omicron XBB1.5 mutant strains (Figures 7A–C). The results showed that DBP had an *IC*_50_ of 49.22 μM for the Delta variant and 53.70 μM for the Omicron XBB1.5 variant. Notably, DBP exhibited stronger inhibitory activity against both variants compared to the WT strain (Figure 7D), suggesting that DBP’s antiviral efficacy is mutation-dependent, consistent with the molecular docking results. Western blot analysis demonstrated that 100 μM DBP effectively inhibited Spike-mediated membrane fusion in both Delta and Omicron XBB1.5 variants. S-protein cleavage was absent in ACE2-negative conditions, confirming that DBP specifically blocks Spike-mediated membrane fusion (Figures 7E–G). These results indicate that DBP has broad-spectrum antiviral activity against multiple SARS-CoV-2 variants.

### 2.8 Site-directed mutagenesis validates Tyr453 and Tyr495 as critical residues for DBP binding

**FIGURE 8:**
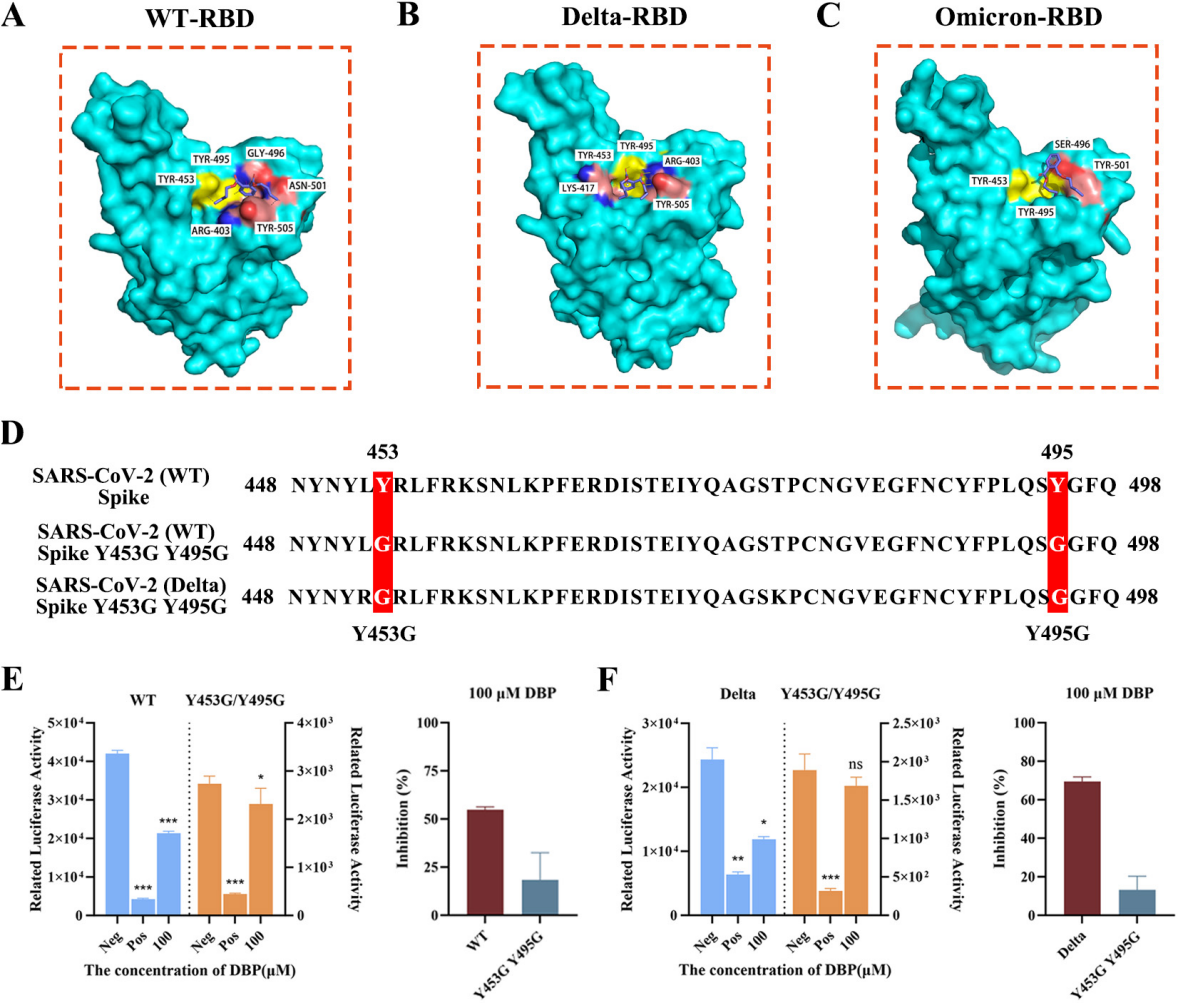
Identification of key residues for DBP-RBD interaction and functional validation of site-directed mutants. **(A–C)** Binding sites of Mutants RBD and DBP, highlighting key interacting residues (Tyr453, Tyr495) with yellow. **(D)** Amino acid sequence alignment of RBD regions (positions 448–498) showing Y453G/Y495G mutations in WT and Delta Spike. **(E,F)** Membrane fusion activity (luciferase assay) and DBP inhibition (%) in WT/E (left: activity; right: inhibition) and Delta/F groups, with mutants (Y453G/Y495G) analyzed. Data: mean ± SD; *P < 0.05, **P < 0.01, ***P < 0.001; ns: no significance.

To elucidate the critical residues mediating DBP-RBD interaction, we performed site-directed mutagenesis targeting Tyr453 and Tyr495, two conserved residues identified from molecular docking analyses (Figures 8A–C; Supplementary Table 3). Structural modeling revealed that Tyr453 and Tyr495 are pivotal for DBP binding across WT, Delta, and Omicron RBDs: WT-RBD forms two hydrophobic interactions with DBP at these sites, Delta-RBD engages in hydrogen bond, π-stacking and hydrophobic interactions, while Omicron-RBD relies on hydrogen bonding networks involving these residues (Table 3). Sequence alignment (Figure 8D) confirmed the conservation of Tyr453 and Tyr495 in SARS-CoV-2 Spike, and we engineered mutants (WT-Y453G Y495G and Delta-Y453G Y495G) to substitute these tyrosines with glycine. Spike-mediated membrane fusion assay (Figures 8E, F) demonstrated that Y453G/Y495G mutations significantly abrogated DBP’s inhibitory efficacy. In the WT background, 100 μM DBP inhibited membrane fusion by 54.831 ± 1.465%, whereas the mutant (WT-Y453G/Y495G) showed a significantly reduced inhibition of only 18.375 ± 14.124% (Figure 8E). A similar trend was observed in the Delta variant: DBP’s inhibition decreased from 69.490 ± 2.365% in the Delta strain to 13.112 ± 7.211% in the Delta-Y453G/Y495G mutant (Figure 8F). Notably, the Y453G/Y495G mutant exerted a more pronounced effect on attenuating DBP’s inhibitory activity in the Delta variant compared to the WT background (Figures 8E, F). These functional results corroborate the structural predictions, confirming that Tyr453 and Tyr495 are essential for DBP’s binding to the RBD and its consequent suppression of Spike-mediated membrane fusion.

**TABLE 3:** Docking results of different mutant strains of RBD with DBP.

RBD mutant strain	PDB ID	Affinity (kcal/mol)	Salt Bridge	Hydrogen Bond	π - Cation	π - Stacking	Hydrophobic
WT-RBD	8DV1	–5.1	/	GLY-496	GLY496	/	ARG-403, Tyr453^▲^, Tyr495^▲^, PHE-497, ASN-501, TYR-505
Delta-RBD	8HRl	–4.8	ARG-403	Tyr453^▲^, ARG-403,	/	Tyr453^▲^	LYS-417, ILE-418, Tyr495^▲^, TYR-505
Omicron-RBD	7WBP	−4.5	/	Tyr453^▲^, Tyr495^▲^, SER-496, TYR-501	SER-496	/	TYR-501

^▲^Conserved sites interacting with DBP.

## 3 Discussion

Although global COVID-19 vaccination has significantly reduced the incidence of severe illness and mortality, persistent mutations in SARS-CoV-2 (e.g., Omicron sublineages and their progeny variants) have diminished the neutralizing efficacy of existing vaccines and monoclonal antibodies, leading to an increased frequency of breakthrough infections (Bruel et al., 2022; Wilhelm et al., 2022; Zhou et al., 2022). Meanwhile, approved antiviral drugs, such as nucleoside analogs like remdesivir, face significant limitations in clinical use. These include the need for intravenous administration, the risk of drug resistance, and high costs, all of which hinder their ability to meet the widespread needs of patients with mild disease (Navacchia et al., 2024; Nhean et al., 2023). Therefore, the development of small molecule drugs that are easy to administer, broad-spectrum, and cost-effective remains a critical global public health. A key step in SARS-CoV-2 infection is the binding of the viral Spike protein to ACE2 receptors on host cells (Wang et al., 2021). This interaction not only determines the efficiency of viral infection but also presents an important target for therapeutic intervention. Blocking the Spike-ACE2 interaction has proven to be an effective approach for early interruption of viral invasion (Xiang et al., 2021).

The strategies of disrupting the Spike-ACE2 interface have motivated extensive efforts to develop small-molecule and peptide-based inhibitors. These inhibitors primarily function through one of two mechanisms: targeting the host ACE2 receptor or the viral Spike RBD (Xiang et al., 2021). Given the critical physiological functions of ACE2, targeting the RBD has emerged as a predominant strategy to minimize potential side effects. Structural studies show that the RBD binds the wedge-shaped α1 helix of ACE2 through a buried surface area (Lan et al., 2020). Consequently, in terms of inhibitory mechanisms, designing peptides that mimic the key segments of ACE2 can act as “molecular decoys” to competitively bind with the viral RBD. For instance, Yu et al. chemically “stapled” a peptide derived from ACE2 residues 21–43, creating a functional mimic of the α1-helix that competitively blocks the RBD binding site (Jiang et al., 2024). In contrast, small molecules are designed to bind with high affinity to critical “hotspot” regions on the RBD, thereby either directly sterically hindering ACE2 access or allosterically inhibiting the formation of key interactions. Ghoula et al. (2023) identified three druggable sites on the SARS-CoV-2 RBD. Sites 1 and 2 are allosteric, located away from the RBD-ACE2 interface, where they function by stabilizing the spike’s “closed” state to indirectly block viral binding. The paramount advantage of site 3, however, is its direct location at the RBD-ACE2 interface. It encompasses critical hotspot residues (K417, N501, Y505), enabling direct, competitive inhibition of viral attachment. Importantly, site 3 aligns with pockets previously reported in independent studies (Dokainish et al., 2022). Linoleic acid has been reported to be able to bind to site1, stabilizing the spike protein in the closed state (Toelzer et al., 2020). GA171 is located at site 3. Its carboxyl group forms a salt bridge with Arg403 and forms a hydrogen bond with Tyr453. The salicylic acid aromatic ring has an edge-to-face π-π stacking interaction with Tyr505 (Xiang et al., 2023). Molecular dynamics indicate that gallic acid (GA) forms hydrogen bonds with G496, N501 and Y505 of S-RBD, and methyl gallate (MG) forms hydrogen bonds with G496 and Y505 (Yu et al., 2024). In this context, this research focuses on the volatile oils of TCM with rich structural diversity and suitable for inhalation administration, aiming to discover Spike-ACE2 inhibitors.

Building on prior previous studies, we analyzed 47 key natural components derived from antiviral essential oils. Using virtual screening and screening using Enzyme-Linked Immunosorbent Assay (ELISA), Caryophyllene oxide (CO), Dibutyl phthalate (DBP), and Patchouli alcohol (PA) were found to significantly inhibit RBD-ACE2 interaction (Figure 2A). In a subsequent SARS-CoV-2 Spike-mediated cell membrane fusion assay, DBP demonstrated the most pronounced inhibitory effect, reducing Spike protein cleavage in a concentration-dependent manner (Figures 3B, 4A). This reduction in cell membrane fusion occurred without broad-spectrum inhibition (Figures 5E–G). The therapeutic index (SI) for membrane fusion inhibition was 4.7. Immunofluorescence co-localization assays further validated these results, suggesting that DBP can inhibit the membrane fusion process mediated by the SARS-CoV-2 Spike protein. To determine whether DBP inhibits the viral endocytosis pathway, a controlled assay using two pseudoviruses, SARS-CoV-2 PsV and VSVΔG/G PsV, was performed. Previous studies have shown that VSVΔG/G PsV, which is based on the HIV-1 lentiviral backbone, enters host cells via the endocytosis pathway (Aiken, 1997; Cabot et al., 2022). In this experiment, DBP inhibited SARS-CoV-2 PsV infection, but had no effect on VSVΔG/G PsV entry. This suggest that DBP does not interfere with viral entry via the endocytosis, but instead specifically blocks the binding of ACE2 to the Spike protein (Figure 3D). Furthermore, this finding was consistently validated using surface plasmon resonance assays.

The time-course ELISA experiment provided crucial functional evidence that DBP exerts its inhibitory effect by binding directly to the RBD. Consistent with this finding, molecular docking revealed that DBP binds stably to the RBD by forming hydrogen bonds with key protein residues, such as TYR-501, Tyr495, SER-496, and Tyr453 (Figure 5E). DBP’s spatial blocking effect caused multiple RBD-ACE2 interface residues (Tyr453, GLY-476, ASN-477, ARG-493, SER-494, ARG-498, THR-500, GLY-502) to lose ACE2 interaction (Figures 5F, G). Notably, DBP also exhibited broad-spectrum inhibitory activity against SARS-CoV-2 variants. As shown in Figure 7D, DBP exhibited the most significant inhibitory effect on the Delta variant, followed by Omicron XBB1.5 and the wild-type (WT). Previous studies have shown that the Delta variant relies more on the membrane fusion for cell entry due to mutations like D614G and P681R (Gong et al., 2021; Starr et al., 2020). We demonstrated that DBP blocks viral entry by inhibiting membrane fusion (Figure 3B), which results in more effective against with the Delta variant. Moreover, the N501Y mutation, known to enhance the binding affinity of SARS-CoV-2 to ACE2, is particularly prominent in multiple variants, including Beta, Gamma, and Omicron (Khatri et al., 2023; Kuzikov et al., 2022). Our findings suggest that TYR-501 is a binding site for DBP on the RBD, and this discovery implies that DBP binds more readily to RBDs carrying the N501Y mutation, which may partially explain its superior inhibitory effect on Omicron XBB1.5 compared to the WT.

The broad-spectrum inhibitory activity of DBP prompted us to investigate its conserved binding sites on the RBD. Molecular docking results revealed that DBP consistently binds to pockets at the RBD–ACE2 interface (Figures 8A–C). In both WT and Delta RBDs, DBP occupies a pocket adjacent to Tyr505 and Arg403. Tyr505 interacts with Lys353 on ACE2 via strong hydrophobic stacking, and is crucial for ACE2 recognition (Xu et al., 2021). Arg403 forms a key salt bridge with Glu37 of ACE2 (Laurini et al., 2020). This binding site overlaps with that of the previously reported inhibitor GA171, suggesting a similar mechanism in which stable binding within this pocket interferes with RBD–ACE2 interaction. However, this finding alone does not explain DBP’s potent inhibitory effect against the Omicron variant, which carries the Y505H mutation. We hypothesize that, although the Y505H substitution disrupts the hydrophobic stacking with DBP, compensatory mutations G496S and N501Y restore binding affinity, allowing DBP to remain anchored in the pocket (Figure 8C). To systematically identify conserved binding residues, we integrated docking poses of DBP with three mutant RBDs, which highlighted Tyr453 and Tyr495 as common interaction sites. Point mutation experiments confirmed their significance (Figures 8E, F). Notably, the mutation of these residues had a more pronounced effect on the Delta variant than on WT. This is likely due to DBP’s binding stability in Delta RBD being more dependent on π-stacking between its phenyl ring and Tyr453 (Figure 8B). Tyr453 forms a direct hydrogen bond (≤ 3.2 Å) with His34 of ACE2 (Yan et al., 2020), emphasizing its potential as a drug target (Guruprasad, 2022). Moreover, Tyr495, located within the critical receptor-binding motif of the spike protein, is essential for high-affinity binding to human ACE2 (Teng et al., 2021; Yang et al., 2021). These interactions between DBP and conserved tyrosine residues are critical for blocking the RBD–ACE2 interaction. Surprisingly, the inhibitors reported at Site 3, such as GA171 and gallic acid derivatives, share common structural features centered on aromatic and carboxylic acid groups (Ghoula et al., 2023; Xiang et al., 2023; Yu et al., 2024). In contrast, DBP possesses a distinct, flexible phthalate scaffold. The novelty of this structural aspect is of great significance, as its “head-leg-hand” configuration enables it to form an optimal binding conformation.

Based on the above insights, we leveraged the Chemical Checker platform^1^ to identify derivatives of DBP from the Approved Drugs library. To further explore structure-activity relationships (SARs), we selected Butyl 4-aminobenzoate, Tetracaine, Oxybuprocaine, and Propoxycaine to represent a progressive structural optimization (Supplementary Figure 1A). Starting from a core aromatic ester with a single amine (Butyl 4-aminobenzoate), we incrementally introduced tertiary amines (Tetracaine), ether oxygens (Oxybuprocaine), and optimized alkyl chains (Propoxycaine). The membrane fusion inhibition rates followed the order: DBP > Propoxycaine > Oxybuprocaine > Tetracaine > Butyl 4-aminobenzoate (Supplementary Figure 1C). This indicates that inhibitory potency is enhanced with targeted structural optimization. Based on this, we propose potential structure optimization directions. First, the benzene ring is essential as a conformational core. Second, introduction of multiple hydrogen bond acceptor (ester O, tertiary amine N, ether O) is critical—these groups can form hydrogen bonds with the partially positively charged H atoms in the phenolic hydroxyl groups of RBD tyrosine residues (Tyr453, Tyr495), strengthening binding stability. Third, compared with oxybuprocaine, ortho-substitution in propoxycaine is more advantageous than meta-substitution. In contrast, the amine group (a hydrogen bond donor providing two H atoms) may interfere with inhibitory activity. For Butyl 4-aminobenzoate—lacking additional hydrogen bond acceptors to compete for RBD binding—its amine group likely forms non-specific hydrogen bonds with ACE2 polar residues, thereby enhancing RBD-ACE2 interactions and promoting membrane fusion (Supplementary Figure 1C). This is consistent with the observation that compounds with balanced hydrogen bond acceptors (Propoxycaine, Oxybuprocaine) avoid such interference and maintain inhibitory activity.

DBP, a widely used plasticizer in the phthalate ester (PAE) group, is present in personal care products (e.g., perfumes, aftershaves, nail care items), children’s toys, pharmaceuticals, and food products. Its primary function is to increase the flexibility of polymers, making them softer and more malleable (Frederiksen et al., 2007; Liu et al., 2016; Wang et al., 2019). However, DBP residues in the environment have raised significant concerns (Latini, 2005). Several studies have highlighted that DBP exhibits reproductive toxicity, immunotoxicity, and endocrine-disrupting effects (Pierozan et al., 2023; Xie et al., 2022; Zhang et al., 2022). Although its toxicity limits direct clinical application, multiple approaches can reduce its toxicity and enhance its antiviral activity, thereby enabling its therapeutic application. First, intranasal administration can enhance therapeutic efficacy while minimizing off-target effects. Emerging clinical evidence shows that prophylactic use of intranasal formulations, including nitric oxide (NO) (Winchester et al., 2021), hydroxypropyl methylcellulose (HPMC) (Shmuel et al., 2021), and azelastine (Meiser et al., 2024), significantly reduces viral load in adult patients with mild COVID-19. This approach targets the nasal mucosa, the site of initial SARS-CoV-2 infection, avoiding systemic exposure (Rajput et al., 2022). Additionally, Further optimization using nanoparticle encapsulation (Deshmukh et al., 2025) may amplify antiviral potency while maintaining safety thresholds. Beyond delivery strategies, Re-designing and modifying the DBP structure can enhance the antiviral activity. For instance, 3-hydroxyphthalic anhydride (3HP)-modified β-lactoglobulin (3HP-β-LG) binds competitively to the RBD of the SARS-CoV-2 spike protein (S-protein), blocking its interaction with the ACE2 receptor. Notably, the inhibitory activity of 3HP-β-LG against SARS-CoV-2 PsV infection increased with higher 3HP modification levels (Hua et al., 2021). Finally, combination therapies involving these modifications, in conjunction with nucleoside analogs (e.g., raltegravir and molnupiravir), offer a practical pathway to clinical application (Schultz et al., 2022).

Despite its antiviral potential, our study has limitations that must be addressed before clinical application. First, current investigations primarily rely on *in vitro* cellular and PsV models. The *in vivo* validation of efficacy and safety—particularly in models of respiratory infection—is currently lacking. Second, while the reproductive and endocrine toxicities of DBP have been confirmed (Aly et al., 2016), the study has not yet optimized the dosage or modified the structure to ensure a therapeutic index suitable for human use. Future studies should prioritize pharmacokinetic profiling and toxicity screens of lead DBP analogs. Notably, replacing the phthalate moiety with bioisosteres could mitigate toxicity while maintaining physical properties. Replacing the phthalic moiety of diethylhexyl phthalate with that of citrate ester results in acetyltributyl citrate, which significantly reduces toxicity (Sung et al., 2020). These steps are critical to advancing DBP-based therapeutics beyond *in vitro* proof-of-concept.

In conclusion, our study provides evidence that DBP, a natural compound derived from TCM volatile oils, functions as a broad-spectrum RBD inhibitor against SARS-CoV-2 variants, including Delta and Omicron XBB1.5. We have systematically elucidated its unique mechanism of action: DBP binds to a conserved epitope on the RBD, with critical residues Tyr453 and Tyr495 identified through site-directed mutagenesis, and acts primarily via a spatially obstructive mechanism to disrupt the Spike-ACE2 interaction. These findings highlight the potential of DBP derivatives, particularly those with optimized structures, as promising candidates for developing broad-spectrum antivirals. The ability of DBP analogs to target evolutionarily stable regions of the RBD, combined with strategies to mitigate toxicity through targeted delivery or chemical modification, positions this class of compounds as a viable framework for combating current and emerging variants.

## 4 Materials and methods

### 4.1 Materials

10 mM/mL Caryophyllene oxide (CO) (HY-N3544), β-caryophyllene (HY-N1415), α-caryophyllene (HY-N6968), Cedarol (HY-N2071), Bisabolol (HY-121222), Nerolidol (HY-N1944), Dibutyl phthalate (DBP) (HY-Y0304), Patchouli alcohol (PA) (HY-N0207) and Pogostone (HY-N1416) were obtained from MedChemExpress and stored at –20 °C for all subsequent experiments. Bergamotene (WKQ-0007133) was purchased from weikeqibiotech. Polybrene and Cell Counting Kit-8 (CCK-8) (GK10001) were purchased from Good Laboratory Practice Blosclence (GLPBIO). Luciferase Assay System with Reporter Lysis Buffer (E4030) was purchased from Promega. NB Transfection Reagent was purchased from bioscien. Hoechst 33,258 was purchased from Solarbio (C0021). SARS-CoV-2 Spike trimer protein (40589-V08H4), ACE2 protein (10108-H08H), Recombinant Anti-ACE2 Antibody (10108-R003), SARS-CoV-2 (2019-nCoV) Spike Antibody (Rabbit PAb) (40592-T62) were purchased from sino biological (Beijing, China). SARS-CoV-2 Spike-ACE2 Interaction Inhibitor Screening Assay Kit (502050) was purchased from Cayman. ACE2 Inhibitor Screening Kit (P0320S) was purchased from Beyotime.

### 4.2 Cells and plasmids

The 293T cell line overexpressing ACE2 (ACE2-293T) (CTCC-009-091) was obtained from MEISENCTCC. The human primary embryonic kidney cell line (293T) (CL-0005) was obtained from procell. The cells were cultured in the Dulbecco’s Modified Eagle Medium (DMEM) media supplemented with 10% FBS at 37 °C in a 55 ± 5% humidity with 5% CO_2_ atmosphere. The SARSCoV-2 spike plasmids of WT, Delta and Omicron XBB.1.5 for cell-cell membrane fusion assay was provided from Guangzhou Laboratory, guangzhou, China. The SARS-CoV-2 Spike pseudovirus and pLV-Spike-C-GFPSpark (VG40589-ACGLN) were purchased from sino biological (Beijing, China). The NL4-3-mCherry-Luciferase and pMD2.G plasmids were purchased from HedgehogBio Science and Technology Ltd.

### 4.3 Virtual screening

Based on the active components of anti-respiratory virus essential oils reported in the literature (Gao et al., 2023), the 3D molecular structure files of each component were retrieved from the PubChem database^2^ and were first converted to PDB format using OpenBabel (O’Boyle et al., 2011). The converted ligand structures were then prepared for docking using AutoDock Tools-1.5.7 (Goodsell et al., 2021). In this step, their rotatable bonds were defined, and hydrogen atoms and Gasteiger charges were added to establish the correct protonation states at physiological pH (7.4). The final prepared structures were saved in PDBQT format. Crystal structures of various conformations of the Spike-ACE2 interaction (PDB IDs: 7TL9, 8C5R, 7TF8, 7XWA) were obtained from the PDB database.^3^ Water molecules and sulfate ions were removed using PyMOL,^4^ followed by hydrogenation and charge assignment for the receptor using the prepare_receptor4 command. Binding sites were predicted using the DoGSiteScorer algorithm available on the Proteins Plus platform.^5^ DoGSiteScorer (Volkamer et al., 2012) is a grid-based method which uses a Difference of Gaussian filter to detect potential binding pockets and splits them into subpockets. Each small molecule was then docked to the protein subpockets with AutoDock Vina-1.2.0 (Eberhardt et al., 2021). The conformations with the strongest binding affinity were selected, and a heatmap was generated using the ComplexHeatmap package in R. Hierarchical clustering was applied to rank the compounds, and the top ten were identified as potential active ingredients that inhibit the interaction between Spike and ACE2.

### 4.4 Cytotoxicity assay

The ACE2-293T cells were plated into 96-well plates at a density of 1.2 × 10^4^ cells/well and incubated overnight. Subsequently, the CO, DBP, or PA solution (at concentrations of 25, 50, 100, 200, 400, or 800μM) was introduced for 24 h. At the conclusion of each experiment, 100 μL of CCK-8 reagent was added to each well, and the cells were further cultured for 2 h at 37 °C. The optical density (OD450) was assessed using a Multiskan microplate reader.

### 4.5 Enzyme-linked immunosorbent assay

In brief, ACE2-His-HRP was added to rFc-RBD-coated test wells containing 50–800 μM of the compounds, as well as to negative control wells without any compounds. Blank wells were left free of both compounds and ACE2-His-HRP. Afterward, HRP-conjugated anti-His antibody and TMB Substrate Solution were added to each well, followed by the addition of HRP Stop Solution. Finally, the plate was read at a wavelength of 450 nm.

### 4.6 ACE2 enzyme activity assay

The ACE2 Inhibitor Screening Kit utilizes fluorescence resonance energy transfer (FRET) technology, which operates on the following principle: MCA serves as a fluorescence donor (Donor), while Dnp functions as a fluorescence acceptor (Acceptor) or quenching group (Quencher). The absorption spectra of these two fluorescent groups partially overlap, and when the distance between them is optimal (typically 7–10 nm), the donor’s fluorescence energy is transferred to the acceptor, resulting in a reduction in the donor’s fluorescence intensity. The ACE2 substrate binds both MCA and Dnp. Upon cleavage of the substrate by ACE2, MCA and Dnp are separated, causing MCA to emit fluorescence. The ACE2-containing assay solution, containing DBP (at concentrations of 0, 12.5, 25, 50, 100, and 200 μM) or the positive control MLN4760 (at concentrations of 20 nM), was prepared. Substrate was then added, followed by mixing. The reaction was incubated at 37°C for 30–60 min, protected from light, and subsequently analyzed by zymography (Ex325nm/Em393nm).

### 4.7 Cell-cell membrane fusion assay

As previously reported (Yu et al., 2022), Human Embryonic Kidney 293T cells (HEK293T) cells were transfected with the SARS-CoV-2 spike (S) protein (WT/Delta/XBB1.5) and Cre plasmid (293T/SARS-CoV-2-S/Cre). Another set of HEK293T cells were transfected with hACE2 and Stop-Luc plasmid (293T/hACE2/Stop-Luc), while only Stop-luc plasmid was transfected in positive control group (293T/Stop-Luc). The cells were cultured in DMEM medium supplemented with 10% FBS at 37°C for 24 h. Following incubation, both cell lines were treated with 0.25% trypsin and then seeded at 80% confluence in a 1:1 ratio. The cells were co-cultured in the presence or absence of DBP at 37°C for 24 h. To assess the inhibition rate, cells were lysed using passive lysis buffer, and luciferase activity was measured using a luciferase reporter system (Promega), following the manufacturer’s protocol. Western blot analysis was performed to evaluate the cleavage of the Spike protein.

For fluorescence co-localization experiments involving cell-cell membrane fusion, HEK293T cells were transfected with both the mCherry and hACE2 plasmids (293T/hACE2/mCherry), while the positive control group was established by transfecting cells with only the mCherry plasmid (293T/mCherry). A separate set of cells was transfected with the Spike-GFP plasmid (293T/Spike-GFP). Cell nucleus were stained with Hoechst 33,258, and subsequent procedures were performed as previously described. Cells were observed and photographed under a fluorescence microscope at 24 h, and the areas of red and yellow overlap were quantified.

### 4.8 SARS-CoV-2 PsV entry inhibition assay

To generate VSVΔG/G Pseudotyped lentiviral particles, 6 × 10^6^ HEK293T cells were plated in a 10 cm dish and cultured overnight. The NB Transfection Reagent (30 μL) was diluted in 250 μL of Opti-MEM. This solution was then combined with a second mixture containing the plasmids NL4-3-mCherry-Luciferase (7.5 μg) and pMD2.G (2.5 μg) in 250 μL of Opti-MEM, according to the manufacturer’s instructions. The combined mixture was incubated for 15 min at room temperature and subsequently added to the plated HEK293T cells. After 6 h, the culture medium was re-placed with fresh complete medium, and the supernatant containing the virus was collected at 48 and 72 h.

A mixture of 25 μL of SARS-CoV-2 PsV or VSVΔG/G PsV in medium was incubated with DBP at concentrations of 0, 25, 50, 75, and 100 μM for 1 h at 37°C. The resulting mixtures (100 μL) were then used to infect ACE2-293T cells. Polybrene with a final concentration of 5 μg/mL was added. After an additional 72 h, cells were lysed using passive lysis buffer, and luciferase activity was measured using a luciferase re-porter system (Promega), following the manufacturer’s protocol.

### 4.9 Surface plasmon resonance assay

Surface plasmon resonance (SPR) assays were conducted using a CM5 sensing chip. The stationary phase was activated with EDC/NHS (1:1 ratio) for 420 s. To characterize the binding of DBP, SARS-CoV-2 Spike S1 + S2 trimer protein and human ACE2 protein were separately immobilized on the experimental channel, while the reference channel was used as a baseline control. Binding and dissociation were recorded at a flow rate of 50 μL/min with a gradient dilution of DBP (starting at 3.125 μM, followed by 4-fold dilutions to ≥ 5 concentrations), for 60 s each. Kinetic parameters, including K_D_, Kon, and Koff, were calculated using either the Kinetic 1:1 Binding model or the Steady-State model. To assess DBP’s inhibitory effect, after im-mobilizing the Spike trimer on the experimental channel, the binding curves for the inhibitor-free group (ACE2 at 250 nM, with 2-fold gradient dilution, 30 μL/min flow rate, 120 s binding, and 80 s dissociation) and the inhibition group (with 100 μM DBP and ACE2-small molecule pre-incubation, 60 s binding and 140 s dissociation) were compared. The difference in binding curves was assessed after regenerating the microarrays with 10 mM glycine-HCl (pH 1.5) for 30 s. DBP’s blocking efficacy on Spike-ACE2 interactions was quantified based on the change in affinity. All experiments were conducted in a 1 × PBS-T buffer system, and data were processed by subtracting the reference channel’s background.

### 4.10 Molecular docking

The 3D structures of ligands were obtained from PubChem^2^ in SDF format and were first converted to PDB format using OpenBabel (O’Boyle et al., 2011). The converted ligand structures were then prepared for docking using AutoDock Tools-1.5.7 (Goodsell et al., 2021), where their rotatable bonds were defined. Hydrogen atoms and Gasteiger charges were added to establish the correct protonation states at physiological pH. The final prepared structures were saved in PDBQT format. Protein as receptors had their PDB files sourced from RCSB Protein Data Bank.^3^ In PyMOL-3.0.0, water and ligands were removed from the protein structure, and PDBQT files were generated. The DoGSiteScorer algorithm (Volkamer et al., 2012) in Proteins Plus^5^ was employed to predict protein pockets. Among the predicted pockets, the one located at the ACE2-RBD binding interface was selected for subsequent docking studies. The center coordinates and dimensions of the selected binding pocket were defined as the docking box. Finally, each protein pocket parameter and the three AutoDock Vina run parameters (energy_range = 5, exhaustiveness = 8, num_modes = 8) were saved in a “config.txt” file. The Lamarckian genetic algorithm ran AutoDock Vina-1.2.0 (Eberhardt et al., 2021) to dock ligands with protein pockets, recording scores and visualizing modes in PyMOL-3.0.0. To determine the docking score of S protein with ACE2, the HDOCK server^6^ was used (Yan et al., 2020). The crystal structures of RBD with and without DBP were uploaded to the server separately. Then, the crystal structure of ACE2 was uploaded for docking analysis. Using the default parameters of the server, the binding scores of the complexes with and without DBP were compared, and the results were visualized in PyMOL.

### 4.11 Site-directed mutagenesis of Spike plasmids

SARS-CoV-2 spike (Wuhan-Hu-1, GenBank: QHD43419.1) was homo sapiens codon-optimized by genescripts and generated *de novo* into pcDNA3.1 vectors by polymerase chain reaction (PCR). Spike WT, Delta and Omicron XBB.1.5 variants containing point and deletion mutations were generated using stepwise mutagenesis using spike construct containing the truncated 19 amino acids at the C-terminal (CTΔ19). Site-directed mutagenesis and deletions were performed using customized primers (synthesized by Sangon) and KOD PLUS neo (TOYOBO) high fidelity polymerase. Parental methylated DNA was digested using *Dpn*I and the resultant PCR products were then transformed into DH5α ultra-competent E. coli (KTSMCC600, AlpaLifeBio). Plasmids were then extracted using DNA extraction miniprep kits (AP-MN-P-250, AxyPrep) and DNA concentrations were adjusted using Nanodrop (Thermo). All mutant plasmids were validated by sequencing service provided by Sangon.

### 4.12 Statistical analyses

GraphPad Prism version 8.0 was used to graph. Statistical analyses were con-ducted using IBM SPSS Statistics software version 24.0. One-way analysis of variance (ANOVA) was performed, followed by the Least Significant Difference (LSD) *post hoc* test for homogeneous variances or the Dunnett T3 test for heterogeneous variances, to assess inter-group differences. Statistical significance is denoted as follows: *P < 0.05, **P < 0.01, ***P < 0.001 relative to the Neg or Mock group; #*P* < 0.05, ##*P* < 0.01, ###*P* < 0.001 relative to the positive control group. The data were presented as mean ± SD, *n* = 3.

## Data Availability

The original contributions presented in this study are included in this article/Supplementary material, further inquiries can be directed to the corresponding authors.

## Footnotes

1
https://chemicalchecker.com/

2
https://pubchem.ncbi.nlm.nih.gov/

3
https://www.rcsb.org/

4
https://pymol.org/

5
https://proteins.plus

6
http://hdock.phys.hust.edu.cn/
