# Correction: Broad-spectrum inhibition of SARS-CoV-2 variants by dibutyl phthalate through allosteric disruption of Spike-ACE2 interface

**DOI:** 10.3389/fmicb.2026.1800393

**Published:** 2026-02-16

**Authors:** Jiafan Chen, Dekuan Guo, Xiaoxuan Guo, Lijun Zhao, Geng Li, Helu Liu, Shaobo Wang, Zizhao Lao, Meiling Zhu

**Affiliations:** 1 Shenzhen Traditional Chinese Medicine Hospital, Shenzhen, Guangdong, China; 2 Shenzhen Baoan Women's and Children's Hospital, Shenzhen, Guangdong, China; 3 State Key Laboratory of Traditional Chinese Medicine Syndrome, Guangzhou University of Chinese Medicine, Guangzhou, China; 4 Shenzhen Clinical College of Integrated Chinese and Western Medicine, Guangzhou University of Chinese Medicine, Shenzhen, Guangdong, China; 5 Chinese Medicine Guangdong Laboratory, Hengqin, Guangdong, China; 6 Guangzhou National Laboratory, Department of Basic Research, Guangzhou International Bio-Island, Guangzhou, China; 7 State Key Laboratory of Respiratory Disease, Guangzhou Laboratory Clinical Base, The First Affiliated Hospital of Guangzhou Medical University, Guangzhou, China

**Keywords:** dibutyl phthalate, SARS-CoV-2, viral entry inhibitor, Spike-ACE2 interaction, cell membrane fusion, site-directed mutagenesis, structure optimization

In the published article, there was an error in the designation of corresponding authors.

The article has three corresponding authors: Shaobo Wang, Zizhao Lao, and Meiling Zhu.

The original version of this article has been updated.

