# Supplementary figures and images for: Broad-spectrum inhibition of SARS-CoV-2 variants by dibutyl phthalate through allosteric disruption of Spike-ACE2 interface
Source: Front Microbiol. 2026 Jan 27;16:1610775. doi: 10.3389/fmicb.2025.1610775 (PMC12889129; doi:10.3389/fmicb.2025.1610775)

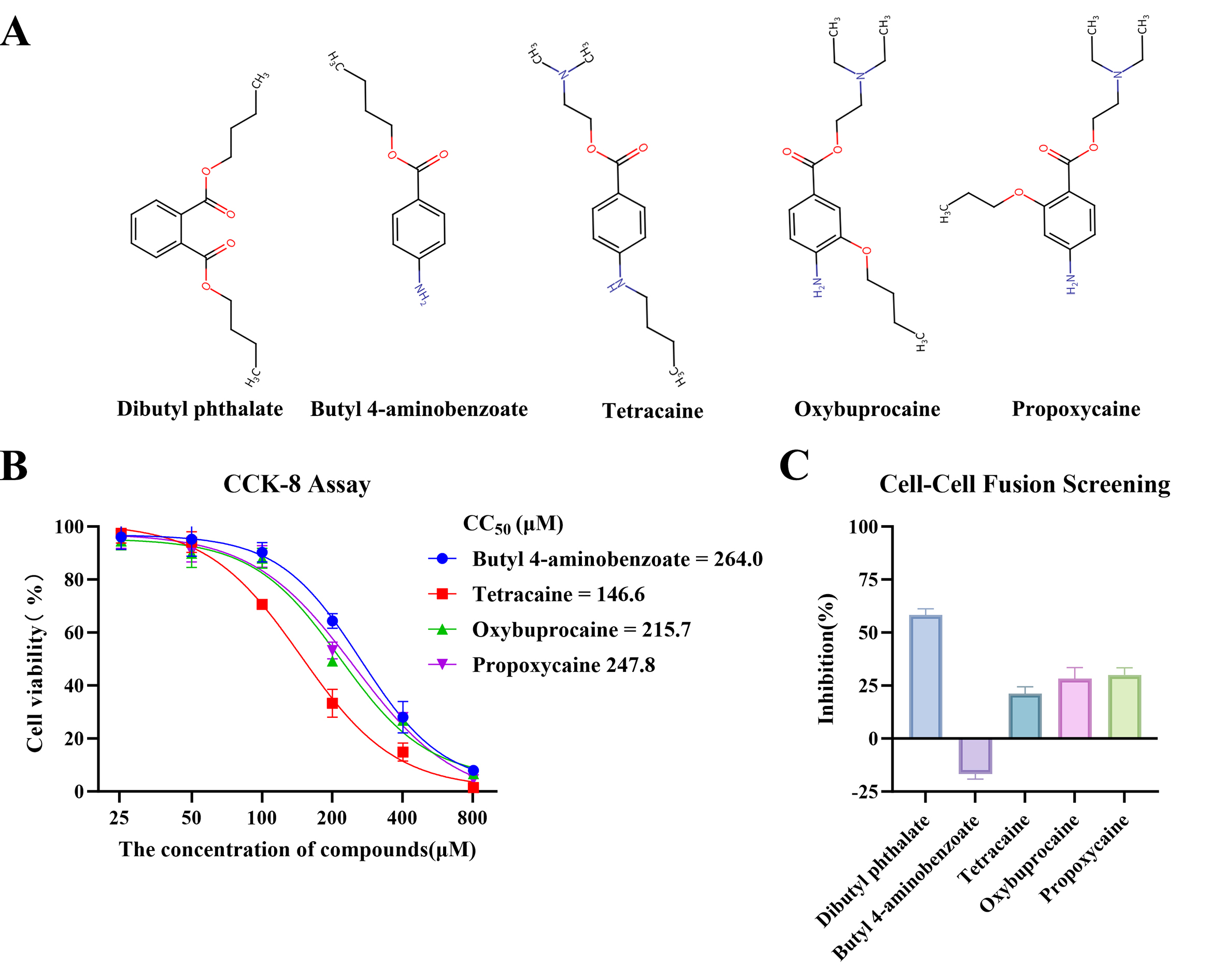

Supplement: Supplementary file 1 [file Image_1.jpeg]
